# Pharmacological Management of Diabetic Macular Edema in Real-Life Observational Studies

**DOI:** 10.1155/2018/8289253

**Published:** 2018-08-28

**Authors:** Laurent Kodjikian, David Bellocq, Thibaud Mathis

**Affiliations:** ^1^Department of Ophthalmology, Croix-Rousse University Hospital, Hospices Civils de Lyon, University of Lyon I, 69004 Lyon, France; ^2^CNRS UMR 5510 Mateis, 69621 Villeurbanne, France

## Abstract

**Objectives of the Study:**

Summary of observational studies concerning the pharmacological management of diabetic macular edema (DME).

**Methods:**

A literature review was conducted using the PubMed database on 1 February 2018 to identify studies evaluating the efficacy of anti-VEGF and dexamethasone (DEX) implants for DME. Studies with more than 10 patients and follow-up of more than 6 months were selected. Analyses were carried out on the overall population and on subgroups defined according to baseline visual acuity (BVA) and the patients' naïve or non-naïve status.

**Results:**

Thirty-two studies evaluating the efficacy of anti-VEGF and 31 studies evaluating the efficacy of DEX-implants were retained, concerning 6,842 and 1,703 eyes, respectively. A mean gain of +4.7 letters for a mean of 5.8 injections (mean follow-up: 15.6 months) and +9.6 letters for a mean of 1.6 injections (10.3 months) was found in the anti-VEGF and DEX-implant studies, respectively. Final VA appears to be similar for both treatment (62 letters for anti-VEGF, 61.2 letters for DEX-implant), and BVA appears lower for DEX-implant, which may partially explain the greater visual gain. The DEX-implant studies show greater gains in VA compared to the anti-VEGF studies, especially for higher BVA. Indeed, mean gains for the subgroups of patients with BVA<50 letters, 50<BVA<60 letters, and BVA>60 letters are +4.3, +5.8, and +3.1 letters, respectively, in the anti-VEGF studies and +10.5, +9.3, and +8.8 letters, respectively, in the DEX-implant studies. Regarding the patient's initial status, only naïve status appears to confer the best functional response in DEX-implant studies.

**Conclusion:**

Observational studies investigating DEX-implant report clinically similar final VA when compared to anti-VEGF, but superior visual gains in real-life practice. This latter difference could be due to the better BVA, but also to the fact that less injections were administered in the anti-VEGF observational studies than in the interventional studies.

## 1. Introduction

Diabetic macular edema (DME) is one of the clinical manifestations of diabetic retinopathy (DR). It is the leading cause of reduced visual acuity and visual impairment in diabetic patients [1, 2]. It develops in approximately 30% of patients who have had diabetes for at least 20 years [3]. Diabetes decreases life expectancy by a mean of 8 years, and 50% of patients with diabetes will die from a cardiovascular event [4]. DME in these patients increases cardiovascular morbidity and mortality by a factor of 2 [5].

The management of DME has changed significantly in recent years. For several decades, laser photocoagulation [6] was the standard treatment for patients with clinically significant DME, leaving macular scars that increase in size over time and can cause secondary vision loss [7]. Since the advent of new pharmacological treatments such as anti-VEGF (vascular endothelial growth factor) and corticosteroids, the management of DME patients has evolved considerably. Intravitreal pharmacological treatments have now become one of the first-line treatments for DME. They come with the benefit of less local side effects, but doubts remain about their possible systemic side effects [8]. Three anti-VEGFs, bevacizumab (Avastin®, Genentech Inc., San Francisco, CA, USA), ranibizumab (Lucentis®, Novartis, Basel, Switzerland), and aflibercept (Eylea®, Bayer, Leverkusen, Germany), and a slow release corticosteroid implant (Ozurdex®, Allergan Inc., Irvine, California) delivering 700 micrograms of dexamethasone (DEX) into the vitreous are used to treat DME. Ranibizumab, aflibercept, and the DEX-implant have been granted European Marketing Authorization (EMA) worldwide for this indication, but this is not the case for bevacizumab. This authorization was obtained based on the data from the pivotal interventional studies. Most of the relevant interventional clinical trials have been performed on carefully selected patient populations. The argument for this strict selection of patients is to ensure that confounding factors do not mask the effect of the treatment. Nevertheless, findings based on a strictly selected patient population cannot be extrapolated to a broader panel of unselected patients. This is a major problem when treatment guidelines are intended for use in routine practice. Indeed, these randomized, controlled studies, with their necessarily stringent inclusion and exclusion criteria, do not obtain data on patients with very high or very low baseline visual acuity (BVA) or with certain comorbidities or on non-naïve patients. In addition, these patients constitute a motivated and observant population; for example, in the pivotal studies patients received a monthly injection over a prolonged time period of 36 months [9, 10]. One can legitimately wonder if the patients treated in routine clinical practice resemble the populations included in the clinical studies. In addition, real-life treatment regimens, i.e., in observational studies, cannot be as stringent as in interventional studies, which often leads to poorer adherence [11, 12]. It is therefore useful to inform our daily practice, not only with the results of interventional studies, but also with the findings of the so-called “real-life” observational studies. The drawbacks of the latter are the potential biases (patients lost to follow-up, missing data) and the lower level of evidence compared to interventional studies. It is therefore vital to consider a significant number of real-life studies, in order to draw valid conclusions.

The objective of this work is therefore to synthetize the available observational studies concerning the pharmacological management of DME.

## 2. Methods

A review of the literature was conducted on the PubMed database on February 1, 2018, to identify all articles investigating the efficacy of anti-VEGF and DEX-implants for treating DME. The key words used were as follows: diabetic macular edema (DME) AND ranibizumab, DME AND Lucentis, DME AND bevacizumab, DME AND Avastin, DME AND aflibercept, DME AND Eylea, DME AND dexamethasone implant, DME AND Ozurdex, DME AND ranibizumab AND aflibercept AND bevacizumab AND dexamethasone implant, and finally DME AND Lucentis AND Avastin AND Eylea AND Ozurdex.

Only articles published in English were selected. Only the ranibizumab, aflibercept, bevacizumab, and dexamethasone implant molecules were retained. Two study designs were found: randomized pivotal studies and observational “real-life” studies. Only the observational studies were selected for this work. Of the studies investigating the efficacy of anti-VEGF and DEX-implant, only series with an initial enrollment of more than 10 patients and follow-up of more than six months were included in the final analysis. For any given study, if different anti-VEGF drugs were used or different types of patients included (for instance naïve versus non-naïve patients), we presented the results separated into different groups of treatment.

The visual acuity (VA) or gain values used for this work were the primary objectives from each study. For the anti-VEGF studies, the VA or gain values used were the end-of-study data, and for the DEX-implant studies, the primary effectiveness endpoints were the maximum mean change in best corrected visual acuity (BCVA) (best improvement) from baseline after each DEX injection. This criterion for DEX-implant was validated by the Food and Drug Administration (FDA) and used in the Reinforce study [13]. VA data expressed in logMAR were converted to the ETDRS score in order to evaluate VA gain or loss relative to the baseline data.

In order to report on functional efficacy, a comparison of VA gains, final VA, and the number of anti-VEGF injections or DEX-implants was initially conducted on the overall study population. Secondary analyses of subgroups, formed according to BVA (less than 50 letters, between 50 and 60 letters, and greater than 60 letters) and the naïve or non-naïve status of the patient at baseline, were also performed. In the case of switching therapy, a minimum wash out time of 1 month was observed in all the studies. The results are presented with the mean gain value and range (minimum and maximum gain observed in studies).

## 3. Results

Our PubMed search initially screened 189 studies, 129 studies of anti-VEGF, and 60 studies of DEX-implants. After eliminating the interventional studies and applying our search criteria (follow-up ≥ 6 months and a minimum of 10 patients included), a total of 32 studies (38 groups of treatment) evaluating the efficacy of anti-VEGF [14–45] and 31 studies (35 groups of treatment) evaluating the efficacy of DEX-implants [13, 26, 46–74] were retained, concerning a total of 6,842 eyes and 1,703 eyes, respectively, for the period between 2005 and 2016.

### 3.1. Overall Population

**Figure 1:**
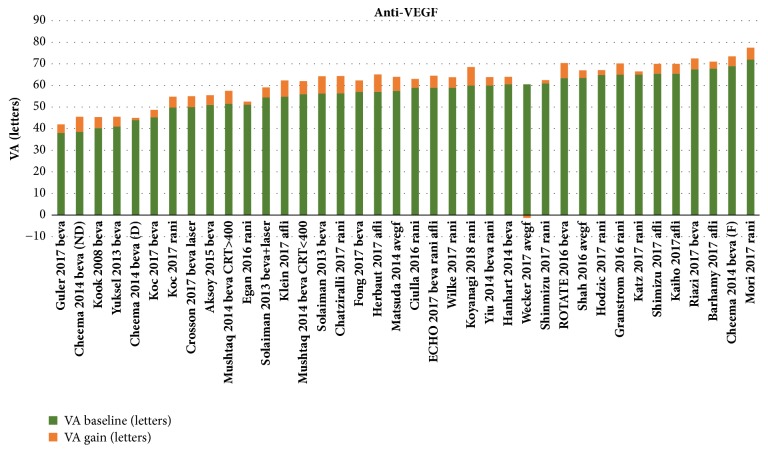
Summary of observational studies investigating the efficacy of anti-VEGF in the treatment of diabetic macular edema.

**Figure 2:**
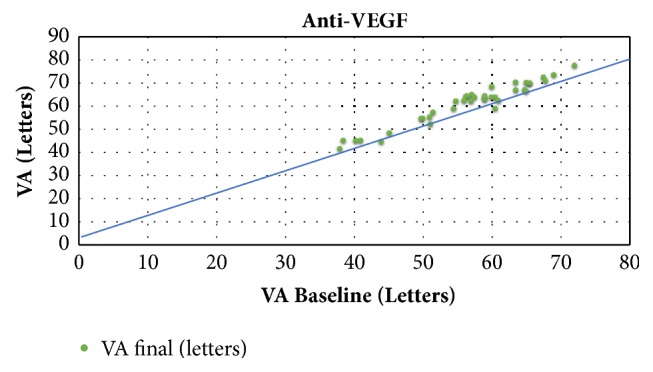
Final visual acuity as a function of baseline visual acuity in studies evaluating the efficacy of anti-VEGF.

For the anti-VEGF studies, patients had a mean BVA of 57.3 letters (range 38-72 letters). Mean follow-up was 15.6 months (6-48 months). During follow-up, a mean gain of + 4.7 letters (-5 - +8.5 letters) (median 4.7 letters) was observed for a mean of 5.8 intravitreal injections (IVI) (1.3-17) (Figures 1 and 2). The mean final VA was 62 letters (42-77.5 letters).  Figure 1 shows the BVA and gain values obtained during follow-up, and the sum of the two which corresponds to final VA, for all of these studies.

**Figure 3:**
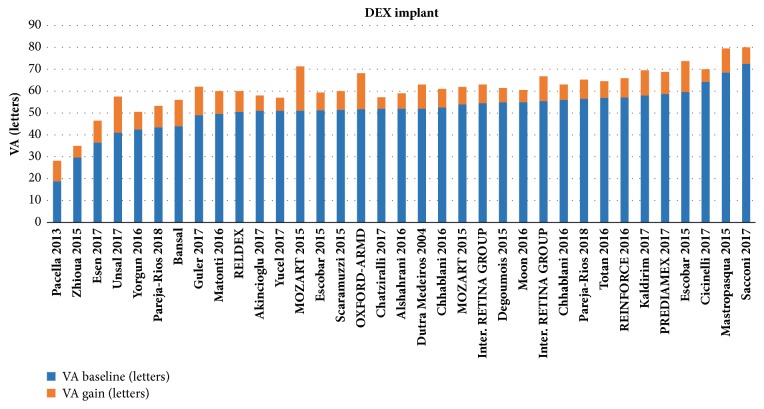
Summary of observational studies investigating the efficacy of the dexamethasone implant in the treatment of diabetic macular edema.

**Figure 4:**
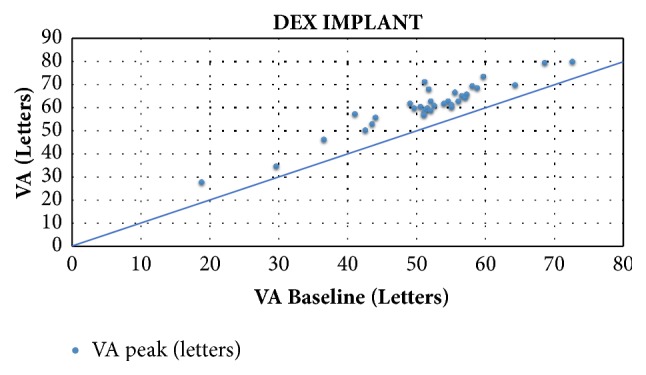
Final visual acuity as a function of baseline visual acuity in studies evaluating the efficacy of the dexamethasone implant.

For the DEX-implant studies, patients had a mean BVA of 51.5 letters (range 18.8-72.5 letters). Mean follow-up was 10.3 months (6-36 months). During follow-up, a mean maximum gain of + 9.6 letters (+5.2 - +20.2 letters) was observed for a mean number of 1.6 IVI (1-3.9) (Figures 3 and 4). The maximum mean VA was 61.2 letters (28.2-80 letters).

**Figure 5:**
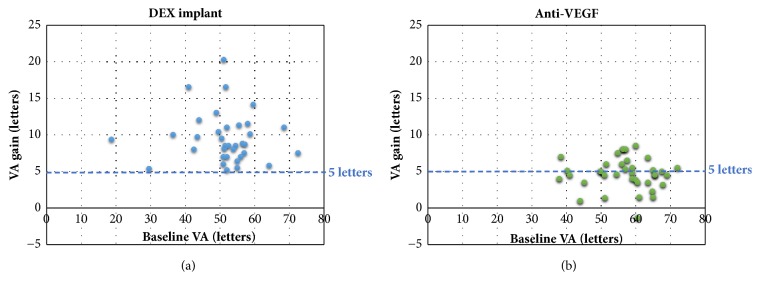
Comparison of mean gain in the different observational studies investigating the efficacy of dexamethasone implant (Figure 5(a)) and anti-VEGF (Figure 5(b)).

Figure 5 illustrates the mean gain on the same scale of visual acuity gains, according to the BVA in the different observational studies assessing the efficacy of anti-VEGF and dexamethasone implants for treating diabetic macular edema.

### 3.2. Segmentation according to the Patient's Baseline Status

By analyzing the results according to the patient's baseline status, we found, in the anti-VEGF studies, a mean gain of + 5 letters for 5.2 IVI in naïve patients (BVA 56 letters) [15, 19, 24, 27, 28, 30, 37] and + 4.8 letters for 6.2 IVI in patients non-naïve to one or more previous treatments (BVA 56.9 letters) [16, 20, 21, 23, 29, 31, 32, 38, 42].

**Figure 6:**
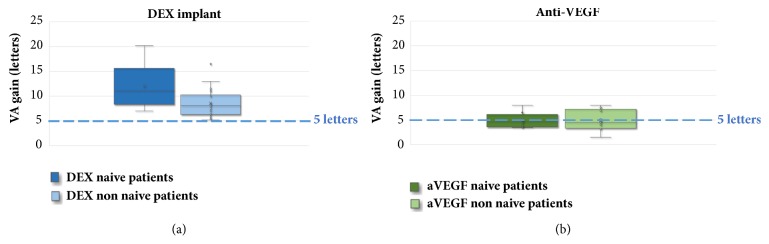
Visual acuity gain in ETDRS letters according to the patients' naïve or non-naïve status in the different observational studies with dexamethasone implant (a) and anti-VEGF (b).

In the DEX-implant studies, there was a mean gain of + 12 letters for 1.9 IVI in naïve patients (BVA 57.9 letters) [50, 54, 56, 59, 62, 65] and + 8.6 letters for 1.4 IVI in non-naïve patients (BVA 48.7 letters) [26, 46, 47, 49–57, 64, 69–73] (Figure 6).

In the anti-VEGF studies, the non-naïve patients had received a mean of at least 10.7 treatments (other anti-VEGF, Triamcinolone IVT, DEX IVT, focal laser) prior to inclusion, compared to 5.4 in the DEX-implant studies.

### 3.3. Segmentation according to BVA

For subgroups with low BVA (<50 letters), there is a mean gain of +4.3 letters in the anti-VEGF studies [26, 28, 29, 32, 34] (mean BVA of 42.4 letters) and +10.5 letters in the DEX-implant studies [48, 55, 56, 61, 65, 66, 71, 73] (mean BVA of 39.4 letters). Mean follow-up was 13 months for a mean of 3 IVI and 9 months for a mean of 1.2 IVI, respectively.

For subgroups with BVA of between 50 and 60 letters, there is a mean gain of + 5.8 letters in the anti-VEGF studies [14, 15, 21, 23–25, 33, 35, 37–39, 45] (mean BVA of 55.7 letters) and + 9.3 letters in the DEX-implant studies [13, 46, 47, 49, 50, 52–54, 56, 57, 61–63, 65–67, 69, 71, 74] (mean BVA of 54.1 letters) with mean follow-up of 16.3 months for a mean of 5.8 IVI and 11.6 months for a mean of 1.75 IVI, respectively.

**Figure 7:**
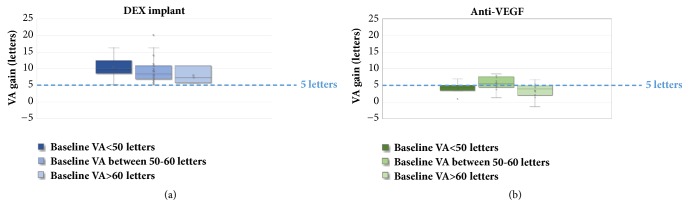
Visual acuity gain (in letters ETDRS) according to the 3 baseline visual acuity subgroups in the observational studies with dexamethasone implant (a) and anti-VEGF (b).

Finally, for subgroups with high BVA (>60 letters), there is a mean gain of + 3.1 letters in the anti-VEGF studies [16–18, 20, 22, 30, 31, 34, 40–42, 44] (mean BVA of 65.3 letters) and + 8.8 letters in the DEX-implant studies [51, 59, 60, 74] (BVA mean of 68.4 letters). Mean follow-up was, respectively, 13.5 months for a mean of 6.5 IVI and 9 months for a mean of 1.8 IVI (Figure 7) (Table 1).

Table 1 summarizes all the results of this study.

## 4. Discussion

Since anti-VEGF and DEX-implants came onto the market, the therapeutic practices for DME have evolved, and laser photocoagulation treatments have gradually been abandoned in favor of IVI. Indeed, the impressive results obtained in clinical trials have encouraged practitioners to use these pharmacological treatments which offer much higher VA gains [9, 10, 75–85] than laser treatment. Indeed, the latter, although it does reduce macular thickness by 50% at 3 years, only rarely improves visual acuity [86]. However, the patients included in the therapeutic trials investigating these pharmacological treatments are selected according to very specific criteria, which do not necessarily correspond to all the patients treated in routine practice. The aim of the present study was to synthetize all the observational studies investigating ranibizumab, aflibercept, bevacizumab, and DEX-implants for DME, in order to evaluate their effectiveness in “real-life” situations.

Indeed, these types of studies have numerous advantages over interventional studies: they provide a more accurate reflection of routine practice; confirm the effectiveness of a treatment under real conditions; include unselected patients under a regimen based on day-to-day practice (actual injection intervals and follow-up); provide complementary data to the interventional studies (notably on the state of practice and possible comparisons between countries); and also provide long-term data. On the other hand, these studies have a number of potential drawbacks, including the possibility of introducing bias (missing data, patients lost to follow-up) and a lower level of evidence. It is therefore essential to analyze a significant number of observational studies and to synthetize their findings, in order to draw scientifically valid conclusions from them.

Real-life observational studies tend to confirm the level of efficacy obtained in interventional studies of pharmaceutical treatments for DME. Indeed, observational studies of anti-VEGF and DEX-implants show VA gains of up to +20 letters. Analysis of the data from these observational studies suggests that the gain in VA is greater with DEX-implant than with anti-VEGF. Indeed, the mean maximum gain obtained after DEX-implant IVI (+9.6 letters) is higher than the mean gain obtained after anti-VEGF (+ 4.7 letters). This increased gain was obtained with a smaller number of IVI in the DEX group compared to the anti-VEGF group. In addition, by setting a gain threshold of 5 letters, the majority of real-life DEX studies achieved increases above this value, in contrast to the majority of anti-VEGF studies (Figure 5).

However, no difference was found in final visual acuity, which is around 62 letters for both groups. This result, which could contradict the observed gains in VA, can be partially explained by the BVA. Indeed, the latter is lower in the DEX studies (BVA 51.5 letters) compared to the anti-VEGF studies (BVA 57.3 letters). This lower level of VA in the DEX studies may relate to the fact that DEX-implant remains a second-line treatment in routine practice [8], thus explaining that the patients included in these studies are mostly non-naïve patients, with a long duration of DME, and therefore lower VA. Regarding the studies selected here, 9/32 anti-VEGF studies only concern non-naïve patients [16, 20, 21, 23, 29, 31, 32, 38, 42] with a mean duration of DME of 30.5 months, compared to 19/31 for DEX studies [13, 26, 46, 47, 49–57, 64, 69–73] with a similar duration of DME (26.8 months). Nevertheless, the mean numbers of treatments are quite different between the anti-VEGF studies and DEX studies with a mean number of 10.7 and 5.4 treatments before switching, respectively, which could constitute a bias.

Thus, in the RELDEX study [67], which is the observational study with the largest number of DME patients included, with a long-term follow-up of 3 years, we found a mean duration of DME of 24.7 months, with only 26.5% naïve patients. This is mainly explained by the fact that the DEX-implant obtained its EMA label later, and the patients included were mostly previously treated with anti-VEGF IVI, focal laser, or triamcinolone IVI. Although non-naïve patients switched to DEX-implants have a gain greater than one ETDRS line, naïve patients treated with this same molecule seem to have much higher gains compared to anti-VEGF, above two ETDRS lines. This difference between naïve and non-naïve patients seems less obvious in anti-VEGF studies. Thus, in the observational studies with anti-VEGF, there do not seem to be any clinically significant differences between naïve and non-naïve patients, with an average gain of + 5 letters for 5.2 IVI and + 4.8 letters for 6.2 IVI.

All these data argue in favor of the earlier use of DEX-implant, either as first-line therapy in naïve patients or more quickly as a second-line therapy. Indeed, in the literature, the response to anti-VEGF seems predictable after 3 to 6 injections [87] and not dependent on the number of injections [88].

Moreover, our analysis of the observational studies shows that patients with low BVA can potentially gain a substantial number of letters. Indeed, Figure 5 also highlights the fact that despite low BVA there are still gains in visual acuity, which are sometimes very significant, and this is especially true in the DEX studies. Indeed, Esen et al. report a maximum gain of + 10 letters in a population whose BVA was 36.5(±9.3) letters [55]. These data show that a functional benefit can be expected even in advanced DME with low BVA. Nevertheless, final VA is ultimately related to baseline VA, which is why it is important to initiate treatment as early as possible: the higher the baseline VA, the higher the final VA (Figures 2 and 4). However, BVA is not the only biomarker and other predictors exist. Indeed, it is clear from Figure 5 that the gains are variable for the same level of BVA. BVA in anti-VEGF and DEX-implant studies is therefore probably not the only explanation for the superior VA gains found in the DEX-implant studies. This assumption is illustrated in Figure 6 which represents the visual acuity gain for each population segment in relation to BVA (less than 50 letters, between 50 and 60 letters, and greater than 60 letters).

In the subgroup with low BVA (<50 letters), there is a marked difference between the anti-VEGF (+4.3 letters) and the DEX-implant (+10.5 letters) studies, whereas BVA is relatively similar (42.4 in anti -VEGF versus 39.4 in DEX-implant studies). This difference is also present in the BVA subgroups between 50 and 60 letters with a mean gain of +5.3 letters and +9.3 letters in the anti-VEGF studies (BVA 57.7 letters) and DEX-implant studies (BVA 54.1 letters), respectively. However, the greatest difference is in the subgroups with BVA of more than 60 letters with a mean gain of +3.1 letters (BVA 65.3 letters) and + 8.8 letters (BVA 68.4 letters), respectively.

Therefore, even taking into account the possibility of a ceiling effect, the difference in gain in favor of DEX-implant in this last subgroup shows that the mean VA gain, which seems better in real life with the DEX-implant, is not only due to the lower mean baseline VA for this molecule, as it persists in the high BVA subgroup (greater than 60 letters).

In comparison to the interventional studies, the observational DEX-implant studies appear to yield better results in terms of VA gain. Indeed, the MAGGIORE study reported a gain of + 2.5L for a mean of 2.9 IVI in the first year [84]. PLACID showed a gain of + 2.4L for 1.7 IVI in the first year [83]. This more limited visual gain in the interventional studies seems to be confirmed at 2 years and 3 years by the BEVORDEX studies (+ 6.9L for 5 IVI at 2 years) [85] and MEAD [82] (+ 2.6L for 4.1 IVI at 3 years). The possibility of retreating at an earlier stage in real life, as opposed to the fixed treatment regimens required for interventional studies (IVI every 6 months for MEAD [82], 5 months for MAGGIORE [84]), and also the large number of non-naïve patients in the pivotal study (duration of DME 23.6 months in MEAD and a low proportion of naïve patients 29.6% [82]) probably explain this difference in results.

On the other hand, the anti-VEGF studies show precisely the opposite, with better results obtained in the interventional studies compared to the observational studies. Indeed, the interventional studies report a higher gain both for studies using a frequent injection schema, such as RESOLVE (gain of + 10.3L for 10.2 IVI in the first year) [77], RISE / RIDE (+ 11.1 letters for 10.7 IVI) [9], or VIVID (gain of + 10.7 letters for the 8.7 IVI group 2q8) and VISTA (gain of + 10.7 letters for 8.4 IVI group 2q8) [10], and for studies using less frequent injection regimens, such as RESTORE (+ 6.8 letters for 7.4 IVI) [79]. This difference in visual acuity gain observed in the observational studies is probably directly related to the number of IVI. Indeed, the mean number of injections in real life is 4.7 in present review, whereas it exceeds a mean of 7 in randomized controlled trials. Indeed, in real life, it is more difficult to monitor and inject a diabetic patient on a monthly basis or more, especially given the well-known issues with compliance, notably during the first years of DME management. These data (VA gain directly related to the number of IVI) seem to be also confirmed for AMD [89–92] and retinal venous occlusions [93, 94]. These results underscore the fact that patients appear undertreated in real-life anti-VEGF studies, and these findings should be explained to the patient at the first consultation to encourage them to accept and schedule a sufficient, high number of injections over the first, and even the second, year of treatment.

Concerning side effects, cataract formation and increasing the intraocular pressure (IOP) are considered to be the main side effects of intravitreal corticosteroids and seem to be dose dependent [95, 96]. Boyer et al. reported in the pivotal interventional study that rates of cataract-related adverse events in phakic eyes were 67.9% and 20.4% for the 0.7 mg DEX-implant and sham groups, respectively. Increased IOP ≥ 25 mmHg was observed in 32.0% and 4.3% for the 0.7 mg DEX-implant and sham groups, respectively, and IOP ≥ 35 mmHg in 6.6% and 0.9% [82].

Some limitations have been also described with anti-VEGF, including the need for frequent injections, induction of resistance, and tachyphylaxis due to the long-term nature of the treatment [97]. Furthermore, sustained hypertension with anti-VGEF has been described since 2010 onwards. One of the reported risk factors is the number of injections [98]. Recently the DCR-net group reported a cumulative probability of sustained IOP elevation or of the initiation or extension of ocular hypotensive therapy by 3 years of 9.5% for the ranibizumab treatment group versus 3.4% for the sham injection treatment group in protocol I in 582 eyes (hazard ratio of 2.9) [99].

Concerning the real-life studies evaluated in present analysis, similar side effects have been reported in anti-VEGF and DEX studies (Table 1). Concerning the anti-VEGF studies, the incidence of cataract formation was between 0% and 15.4% [14, 20, 21, 24, 26, 28, 41, 42]. None of the studies reported statistically significant elevation of IOP in anti-VEGF studies, but the mean follow-up was only 15 months. However, only Blinder et al. reported an elevation over 10 mmHg in 5.1% of patients [14]. Regarding DEX studies, the incidence of cataract formation was between 0% and 50% [26, 46–74], depending on the number of injections [67]. Concerning IOP, an increase was reported between 0% and 29.5% of studies. The RELDEX study, analyzing 128 DME eyes, used a very strict definition (IOP > 25 mmHg) and found a rate of 10.2% [67]. Pareja-Rios et al., analyzing 113 DME eyes, found a rate of 4% (rise of IOP > 10 mmHg) [65]. Güler logically reported significantly higher IOP elevation in the DEX group than the anti-VEGF one (P<0.001) [26]. Only one patient in the 31 articles analyzed required filtering surgery (0.058%). The SAFODEX study [100] evaluated the tolerance of DEX-implant in 421 consecutives eyes which received 1,000 injections. Ocular hypertension was recorded for 28.5% (120 eyes) of injected eyes over a mean follow-up period of 16.8 months [3–55], but the risk of hypertension in DME eyes was statistically significantly inferior to that in uveitis and RVO eyes. Out of these 120 hypertonic eyes, intraocular pressure lowering medication was required for 31% of eyes, only three patients (affected by RVO) required filtering surgery, and, most importantly, topical treatment alone was sufficient for 97% of the cases. These results demonstrate that safety is easily manageable. Finally, the data are difficult to stack because the definitions of hypertension and the IOP threshold for drug prescription in the different studies are not the same (Table 2).

Lastly, only one endophthalmitis has been reported in the 31 articles among 2897 injections that have been realized (0.03%).

Our analysis has several limitations including the fact that study data searches on PubMed are not always capable of identifying all relevant material. Moreover, it is methodologically imperfect to make indirect comparisons between studies with different numbers, even if this provides an overall vision of the real-life data. 6,842 eyes were included in the anti-VEGF studies versus 1,703 eyes with the dexamethasone implant. Moreover, we report definitions of gain in VA for anti-VEGF studies different from those reported for DEX-implant studies. Indeed, for the anti-VEGF studies, the VA or gain values used were the end-of-study data, but for the DEX-implant studies, the VA or gain values used were the maximum mean change in BCVA (best improvement) from baseline after each DEX injection. This criterion for DEX-implant was validated by the FDA [13], because when DEX-implants are administered, less injections are required, so if final VA is measured a long time after the last injection, it is more likely to be underestimated. In addition, cataracts may form with DEX-implants, and thus artificially reduce the patient's vision. Finally, the best VA criterion corresponds to the primary outcome measure for the vast majority of the DEX-implant studies analyzed. Lastly, the results for the three different anti-VEGFs were taken together as a whole. In some studies, such as protocol T [81], some differences in efficacy were found between the three drugs, which might influence or explain some of the data in the present study.

By definition, observational real-life studies have limitations with patients lost to follow-up and missing data. However, the way that missing data are handled is not always reported in real-life studies and is variable and heterogeneous across the different articles. Nevertheless, these studies are primordial because they complete the pivotal studies because the patients included in “real-life” studies are not subject to selection and correspond to the patients seen in our daily routine practice and the treatment regimen corresponds to “real-life” injection intervals and “real-life” follow-up.

An additional potential bias is the difference in the proportion of naïve and non-naïve eyes: one-fifth of the Ozurdex eyes were treatment naïve versus two-thirds of the anti-VEGF eyes.

In conclusion, pharmacological treatment with anti-VEGF and DEX-implant shows significant VA gains in observational studies. The DEX-implant results report clinically VA gains that appear to be better than real-life gains from anti-VEGF. This impression of greater efficacy may be due to the lighter treatment regimen for this molecule and also to its specific mode of action. The choice of treatment must, however, take into account each individual patient's characteristic in order to offer them personalized treatment.

## Figures and Tables

**Table 1:** Summary of BVA, gain, final VA and mean number of injections in the overall population and subgroups for anti-VEGF and DEX-implant observational studies (BVA: baseline visual acuity, IVI: intravitreal injection.

	Anti-VEGF							DEX-implant				
	Number of eyes	BVA (letters)	Mean gain (letters)	Final VA (letters)	Mean follow-up (months)	Mean IVI	Number of eyes	BVA (letters)	Mean gain (letters)	Max VA (letters)	Mean follow-up (months)	Mean IVI
Overall population	6842	57.3	+4.7	62	15.6	5.8	1703	51.5	+9.6	61.2	10.3	1.6
BVA												
≤ 50 letters	449	42.4	+4.3	46.7	13	3	363	39.4	+10.5	49.9	9	1.2
50-60 letters	4773	55.7	+5.8	61.7	16.3	5.8	1218	54.1	+9.3	63.7	11.6	1.75
≥ 60 letters	1620	65.3	+3.1	68.3	13.5	6.5	122	68.4	+8.8	76.5	9	1.8
Initial Status												
Naive patients	781	56	+5	61	12.3	5.2	176	57.9	+ 12	69.9	10.8	1.9
Non-naïve patients	413	56.9	+ 4.8	61.8	12.1	6.2	801	48.7	+8.6	57.3	9.2	1.4

**Table 2:** Summary of anti-VEGF and DEX-implant studies (VA: Visual Acuity, CSMT: central subfield macular thickness, ND: nondeclared, IVI: intravitreal injection, IOP: intraocular pressure).

Study	Drugs	Study design	Patient status	Number (eyes)	Follow-up (months)	Mean Number IVI	Baseline VA (letters)	Final VA (letters)	Mean VA gain (letters)	IOP	Cataract progression/extraction	Others complications
Blinder KJ et al. [14]	Anti-VEGF	Retrospective	Mixed	156	36	14.2	59.0	64.5	5.5	7.7% IOP ≥ 25 mmHg, 1.3% IOP ≥35 mmHg, rise of IOP ≥10 mmHg in 5.1%	15.4%	No endophthalmitis
Matsuda S et al. [15]	Anti-VEGF	Retrospective	Naïve	124	12	5.8	57.5	64	6.5	ND	ND	ND
Shah CP et al. [16]	Anti-VEGF	Prospective	Non-Naïve	30	29.2	17.0	63.5	67	3.5	ND	ND	ND
Shimizu N et al. [17]	Anti-VEGF	Retrospective	Mixed	46	6	2.7	65.5	70	4.5	ND	ND	ND
Wecker T, et al. [18]	Anti-VEGF	Retrospective	ND	479	12	17.0	60.5	59.2	-1.3	ND	ND	ND
Yiu G et al. [19]	Anti-VEGF	Retrospective	Naïve	33	6	2.7	60	64	3.85	ND	ND	ND
Barhamy B et al. [20]	Aflibercept	Prospective	Non-Naïve	43	6	5.0	67.8	71	3.2	0% (IOP ≥ 25mmHg or a rise of IOP ≥ 10mmHg)	0%	None
Herbaut A. et al. [21]	Aflibercept	Retrospective	Non-Naïve	29	6	3.0	57.1	65.1	8	ND	ND	ND
Kaiho T et al. [22]	Aflibercept	ND	Non-Naïve	51	12	3.8	65.5	70	4.5	ND	ND	ND
Klein et al. [23]	Aflibercept	Retrospective	Non-Naïve	11	6	4.7	54.8	62.3	7.5	ND	ND	ND
Aksoy S et al. [24]	Bevacizumab	Prospective	Naïve	20	6	6.0	51.0	55.5	4.5	10% (IOP > 21mmHg)	2.50%	None
Fong DS et al. [25]	Bevacizumab	Retrospective	Mixed (65% Naïve, 30% Laser, 4% Steroïd)	309	24	3.1	57	62.3	5.3	ND	ND	ND
Güler E et al. [26]	Bevacizumab	Prospective	ND	20	9	6.0	38	42	4	ND	0%	None
Hanhart J et al. [27]	Bevacizumab	Retrospective	Naïve	35	8	3.6	60.5	64	3.5	ND	ND	ND
Koc C et al. [28]	Bevacizumab	Retrospective	Naïve	90	24	4.9	45.2	48.7	3.5	ND	13.7%	1 cerebrovascular accident
Kook D et al. [29]	Bevacizumab	Prospective	Non-Naïve	126	12	2.7	40.3	45.4	5.1	ND	ND	ND
Riazi-Esfahani M et al. [30]	Bevacizumab	ND	Naïve	46	6		67.5	72.5	5	6.5% (IOP ≥ 21 mmHg)	0	ND
Fechter BS et al. [31]	Bevacizumab	ND	Non-Naïve	30	12	9.3	63.5	70.35	6.9	ND	ND	None
Yuksel E et al. [32]	Bevacizumab	Retrospective	Non-Naïve	71	9.8	2.0	41	45.5	4.5	ND	ND	ND
Solaiman KAM et al. [33]	Bevacizumab	Prospective	ND	22	14	3.3	56.3	64.3	8	ND	9.1%	Subconjunctival haemorrhage
Cheema HR et al. [34]	Bevacizumab (Diffuse)	Retrospective	ND	28	6	1.3	44.0	45	1	ND	ND	ND
Cheema HR et al. [34]	Bevacizumab (Focal)	Prospective	ND	20	6	2.1	69.0	73.5	4.5	ND	ND	ND
Cheema HR et al. [34]	Bevacizumab (NSD)	Retrospective	ND	13	6	2.6	38.5	45.5	7	ND	ND	ND
Mushtaq B et al. [35]	Bevacizumab CSMT<400	ND	ND	81	12	3.3	56	62.5	6	ND	ND	ND
Mushtaq B et al. [35]	Bevacizumab CSMT>400	ND	ND	94	12	4.0	51.5	57.5	6	ND	ND	ND
Crosson JN et al. [36]	Bevacizumab	Retrospective	ND	102	60	5.5	50	55	5	ND	ND	ND
Solaiman et al. [33]	Bevacizumab	Prospective	ND	22	14	2.4	54.5	59.1	4.6	ND	9.1%	ND
Chatziralli I et al. [37]	Ranibizumab	Retrospective	Naïve	332	12	6.7	56.4	64.4	8	ND	ND	ND
Ciulla TA et al. [38]	Ranibizumab	Retrospective	Non-Naïve	33	12	6.0	59.0	63	4	ND	ND	ND
Egan C, et al. [39]	Ranibizumab	Retrospective	Mixed (49,6% Naïve)	3103	24	5.4	51.1	52.5	1.4	ND	ND	1 endophthalmitis
Granstrom T et al. [40]	Ranibizumab	Retrospective	ND	59	12	5.0	65	70.2	5.2	ND	ND	ND
Hadzibegovic DH et al. [41]	Ranibizumab	Retrospective	Mixed (97% Naïve)	566	48	13.5	64.8	67.1	2.3	ND	9.9%	1 traumatic cataract
Katz G et al. [42]	Ranibizumab	ND	Non-Naïve	40	16	8.4	65	66.5	1.5	ND	0	None
Koc C et al. [28]	Ranibizumab	Retrospective	Naïve	101	24	6.9	49.8	54.8	5	ND	7.1%	None
Koyanagi Y et al. [43]	Ranibizumab	Retrospective	Non-Naïve	25	12	7.4	60	68.5	8.5	ND	0	None
Mori Y et al. [44]	Ranibizumab	Retrospective	Non-Naïve	68	12	6.4	72	77.5	5.5	ND	ND	ND
Wilke RGH et al. [45]	Ranibizumab	Retrospective	ND	335	36	10.0	59	63.8	4.8	ND	ND	ND
Akincioglu D et al. [46]	DEX Implant	Retrospective	Non-Naïve	57	12	1.3	51	58	7	28% (rise of IOP > 10 mmHg)	21.5%	ND
Alshahrani ST et al. [47]	DEX Implant	Retrospective	Non-Naïve	26	6	1.0	52	59	7	26% IOP > 21mmHg	1.8%	ND
Bansal P et al. [48]	DEX Implant	Retrospective	Mixed	67	6	1.0	44	56	12	12% (IOP > 21 mmHg)	4.5%	Subconjunctival haemorrhage
Chatziral.li I et al. [49]	DEX Implant	Prospective	Non-Naïve	54	12	2.1	52		5.2	5.6% (IOP > 20mmHg)	4.3%	ND
Chhablani J et al. [50]	DEX Implant	Retrospective	Non-Naïve	64	7.67	1.3	52.5	61	8.5	7.6% (rise of IOP > 10 mmHg)	2.5%	ND
Chhablani J et al. [50]	DEX Implant	Retrospective	Naïve	15	11	1.3	56	63	7	7.6% (rise of IOP > 10 mmHg)	2.5%	ND
Cicinelli MV et al. [51]	DEX Implant	Retrospective	Non-Naïve	45	12	1.9	64.2	70	5.8	18.4% (IOP ≥ 20 mmHg)	20%	ND
Degoumois A et al. [52]	DEX Implant	Retrospective	Non-Naïve	42	20.6	1.6	55	61.4	6.4	8% (IOP > 25 mmHg), 2 % (IOP> 30 mmHg)	4.8%	ND
Dutra Medeiros M et al. [53]	DEX Implant	Retrospective	Non-Naïve	58	6	1.0	52	63	11	No anecdotal IOP elevation	ND	None
Escobar-Barranco JJ et al. [54]	DEX Implant	Prospective	Non-Naïve	40	6	1.9	51.3	59.4	8.1	7.9% (rise of IOP > 10 mmHg)	2.6%	3.9% Intravitreal haemorrhage
Escobar-Barranco JJ et al. [54]	DEX Implant	Prospective	Naïve	36	6	1.9	59.6	73.6	14.1	7.9% (rise of IOP > 10 mmHg)	2.6%	3.9% Intravitreal haemorrhage
Esen E et al. [55]	DEX Implant	Retrospective	Non-Naïve	25	6	1.0	36.5	46.5	10	16% (IOP > 21 mmHg)	4%	None
Güler E et al. [56]	DEX Implant	Prospective	Non-Naïve	15	6		49	62	13	20%	0	None
Iglicki et al. [57]	DEX Implant	Retrospective	Non-Naïve	59	24	3.1	54.5	63	8.5	7.1%	ND	ND
Iglicki et al. [57]	DEX Implant	Retrospective	Naïve	71	24	3.9	55.5	66.8	11.3	11.4%	ND	ND
Kaldirim H et al. [58]	DEX Implant	Retrospective	Non-Naïve	35	6	1.0	58	69.5	11.5	11.4%	0%	ND
Lozano Lopez V et al. [59]	DEX Implant	Retrospective	ND	36	6				10.9	29.5% (IOP > 23 mmHg) 1.1% filtering surgery	ND	None
Mastropasqua R et al. [60]	DEX Implant	Prospective	Naïve	27	6	1.7	68.5	79.5	11	0%	0%	ND
Matonti F et al. [61]	DEX Implant	Retrospective	Mixed	23	12	2.1	49.6	60	10.4	11.7% (IOP> 25 mmHg)	0%	26% Subconjunctival haemorrhage
Moon BG et al. [62]	DEX Implant	Retrospective	Mixed	186	6	>1	55	60.5	5.5	4.3% (IOP > 30mmHg)	23.2%	1 Infectious endophthalmitis
Guigou S et al. [63]	DEX Implant	Retrospective	Naïve	16	6	1.2	51.1	71.3	20.2	11.7% (IOP > 25 mmHg), 13.3% (rise of IOP > 10 mmHg)	0%	26% Subconjunctival haemorrhage, 8.6% Intravitreal haemorrhage
Guigou S et al. [63]	DEX Implant	Retrospective	Mixed (20,5% De Naïve)	78	6	1.2	53.9	61.92	8	11.7% (IOP > 25 mmHg), 13.3% (rise of IOP > 10 mmHg)	0%	27% Subconjunctival haemorrhage, 2.6% Intravitreal haemorrhage
Aknin I et al. [64]	DEX Implant	Retrospective	Mixed	29	18	1.5	51.7	68.2	16.5	6.9% (IOP > 25 mmHg)	13.8%	None
Pacella E et al [65]	DEX Implant	ND	Non-Naïve	20	6	1.0	18.8	28.15	9.4	5.8% (IOP >26 mmHg)	0%	None
Pareja-Rios et al. [66]	DEX Implant	Retrospective	Naïve	113	12	1.4	43.5	53.2	9.7	4% (rise of IOP > 10mmHg)	ND	None
Pareja-Rios et al. [66]	DEX Implant	Retrospective	Naïve	11	12	1.4	56.5	65.3	8.8	4% (rise of IOP > 10mmHg)	ND	ND
Bellocq D et al. [67]	DEX Implant	Prospective	Mixed (73% Naïve)	37	6	1.5	58.7	68.7	10.1	14% (IOP > 25 mmHg), 3% (IOP > 35mmHg) 8% (rise of IOP > 10 mmHg)	ND	Subconjunctival haemorrhage
Fine et al. [13]	DEX Implant	Prospective	Non-Naïve	101	12	2.0	57.2	65.9	8.7	12.2% (IOP > 25 mmHg), 2.8% (IOP > 35mmHg) 12.8% (rise of IOP > 10 mmHg)	ND	Vitreous floaters (4.3%)
Mal.clès A et al. [68]	DEX Implant	Retrospective	Mixed (27% Naïve)	128	36	3.6	50.5	60.6	9.5	10.2% (IOP > 25 mmHg), 2.3% (IOP > 35mmHg) 19% (rise of IOP > 10 mmHg)	ND	ND
Sacconi R et al. [69]	DEX Implant	Prospective	Mixed	14	12	1.7	72.5	80	7.5	21% (IOP > 21 mmHg)	0%	ND
Scaramuzzi M et al. [69]	DEX Implant	Retrospective	Mixed (7% Naïve)	15	12	2.0	51.5	60	8.5	20%	8.3%	ND
Totan Y et al. [70]	DEX Implant	Prospective	Non-Naïve	30	6	>1	57	64.5	7.5	13.3% (IOP > 21 mmHg)	0%	ND
Unsal. E et al. [71]	DEX Implant	Retrospective	Non-Naïve	46	6	1.1	41	57.5	16.5	17.4% (IOP > 25 mmHg)	8.7%	12% Subconjunctival haemorrhage
Yucel OE et al. [72]	DEX Implant	Retrospective	Non-Naïve	30	BVA (letters)	1.0	51	57	6	16.7% (IOP > 23 mmHg)	13%	None
Zhioua I et al. [73]	DEX Implant	Retrospective	Non-Naïve	13	9	1.1	29.6	35	5.4	15.4% (IOP > 21 mmHg)	7.9%	None
Yorgun MA et al. [75]	DEX Implant	Retrospective	Non-Naïve	41	6	1.0	42.5	50.5	8	12% (IOP> 21 mmHg)	0%	None
